# Retraction on “Dihydromyricetin attenuates inflammation through TLR4/NF-kappa B pathway”

**DOI:** 10.1515/med-2021-0331

**Published:** 2021-08-13

**Authors:** Nianshui Jing, Xinnan Li

**Affiliations:** Department of Pharmacy, Jinan No. 2 People’s Hospital, Jinan 250001, Shandong, China

**Retraction on:** Nianshui Jing, Xinnan Li. Dihydromyricetin attenuates inflammation through TLR4/NF-kappa B pathway. Open Med. 14(1). doi: 10.1515/med-2019-0083.

This article has been retracted, as there is high risk that it presents images that have been manipulated, which suggests that the results could be fabricated.

